# Acute Leukemia Induces Senescence and Impaired Osteogenic Differentiation in Mesenchymal Stem Cells Endowing Leukemic Cells with Functional Advantages

**DOI:** 10.1155/2019/3864948

**Published:** 2019-04-01

**Authors:** Ximena Bonilla, Natalia-Del Pilar Vanegas, Jean Paul Vernot

**Affiliations:** ^1^Cellular and Molecular Physiology, Faculty of Medicine, Universidad Nacional de Colombia, Bogotá D.C. 111321, Colombia; ^2^Biomedical Research Institute, Faculty of Medicine, Universidad Nacional de Colombia, Bogotá D.C. 111321, Colombia

## Abstract

Mesenchymal stem cells (MSC) constitute an important cell population of the bone marrow hematopoietic niche that supports normally hematopoietic stem cells (HSC) but eventually also leukemic cells. The alterations that occur in the MSC under leukemic stress are not well known. To deepen on this topic, we have used an *in vitro* model of the leukemic niche (LN) by coculturing MSC with an acute lymphocytic leukemia cell line (REH) and proceeded to evaluate MSC characteristics and functions. We found that leukemic cells induced in MSC a significant increase both in senescence-associated *β*-galactosidase activity and in p53 gene expression. MSC in the LN also showed a persistent production of cytoplasmic reactive oxygen species (ROS) and a G2/M phase arrest of the cell cycle. Another acute leukemic cell line (SUP-B15) produced almost the same effects on MSC. REH cells adhere strongly to MSC possibly as a result of an increased expression of the adhesion molecules VCAM-1, ICAM-1, and CD49e in MSC and of CD49d in REH cells. Although mesensphere formation was normal or even increased, multipotent differentiation capacity was impaired in MSC from the LN. A REH-conditioned medium was only partially (about 50%) capable of inducing the same changes in MSC, suggesting that cell-to-cell contact is more efficient in inducing these changes. Despite these important effects on MSC in the LN, REH cells increased their cell adhesion, proliferation rate, and directed-migration capacity. In conclusion, in this *in vitro* LN model, leukemic cells affect importantly the MSC, inducing a senescence process that seems to favour leukemic cell growth.

## 1. Introduction

The bone marrow (BM) niche [1, 2] is an important compartment for the maintenance and regulation of hematopoietic stem cell (HSC) function, i.e., self-renewal, differentiation capacity, and cell migration [3, 4]. Although complex, niche cues are essential for ensuing a functional hematopoiesis during homeostasis and in stressful conditions. This niche encompasses different cell types, including stromal cells of mesenchymal or hematopoietic origin (including immune cells and their progenitors), extracellular matrix components, soluble factors, and sympathetic nerve fibers [3]. In particular, mesenchymal stem cells (MSC) in the niche have been proposed as essential mediators in the maintenance and function of HSC [5, 6]. Different surface molecules and soluble factors are involved in HSC homing, adhesion, and maintenance (mainly, VCAM-1, CD44, LFA-1, c-kit, CXCR4, SDF-1, and SCF) [7, 8].

Many studies have shown that during leukemia proliferation, the hematopoietic niche is remodeled, altering its properties by mechanisms that are only partially understood, but may include abnormal expression of cell adhesion molecules, aberrant migration capacity, and secretion of soluble factors, among others [9–12]. It is believed that these changes improve the survival and proliferation of leukemic cells in the niche [13] to the detriment of HSC [10, 14]. Specifically, the information related to MSC alterations in the leukemic microenvironment, and the molecular mechanisms involved, is scarce with some exceptions in AML and CML [15–18]. Interestingly, it has been described that MSC obtained from multiple myeloma patients exhibited senescence features including a decrease in cell proliferation, loss of osteogenic differentiation potential, and increase in soluble factor secretion [12, 19]. In the same way, a defective osteogenic differentiation was observed in CML patients and cell lines [17] and stromal cell and osteoblast degradation was also reported in AML [18]. Also, in mouse models of Notch-1-induced T-ALL, it has been shown that cell proliferation capacity and differentiation potential of MSC were reduced due to cellular senescence, affecting mainly hematopoietic progenitor cells (HPC) [10].

Cellular senescence is defined as a process in which cells enter an irreversible cell cycle arrest maintaining a metabolic activity with the production of the so-called senescence-associated secretory phenotype (SASP) [20, 21]. Cellular senescence is induced by different types of cell injury, including telomeric shortening, genomic instability, oxidative stress, oncogene expression, and chronic inflammation [22, 23].

We have recently established an *in vitro* leukemic niche (LN) model to simulate BM cell interactions and to study functional alterations of HSC in a leukemic microenvironment [14, 24]. In the present work, we found that leukemic cells induce MSC senescence by a p53-mediated pathway and ROS production. MSC stemness functions were also partially affected. MSC alterations were only partially reproduced with a leukemic-conditioned media, highlighting the relevance of MSC and leukemic direct cell contact. In this senescent microenvironment with reduced stemness function, leukemic cells improved their performance in terms of cell adhesion, proliferation, and migration. Altogether, these results gave us meaningful information about potential therapeutic targets to avoid leukemic cell survival and drug resistance in the BM microenvironment.

## 2. Materials and Methods

### 2.1. BM Mesenchymal Stem Cell Isolation and Characterization

MSC were isolated from femoral BM aspirates of healthy paediatric donors who consulted for traumatic events. Samples were obtained after the approval of their parents by signed informed consent. The Ethical Committee of the Faculty of Medicine, Universidad Nacional de Colombia, approved the protocols. Samples were collected in a sterile tube containing 0.25% EDTA in PBS 1x (GIBCO-Invitrogen, Grand Island, NY, USA). Next, mononuclear cells were isolated by Ficoll density gradient centrifugation (Histopaque *d* = 1.077 g/cm^3^, Sigma-Aldrich, St. Louis, MO, USA) and plated at a density of 10^6^ cells/cm^2^ in Iscove's modified Dulbecco's medium (IMDM) GlutaMAX-I (GIBCO-Life Technologies, Grand Island, NY, USA) supplemented with 1% sodium pyruvate (GIBCO-Life Technologies, Grand Island, NY, USA), 1% minimum essential medium (MEM) nonessential amino acid solution 100x (GIBCO-Life Technologies, Grand Island, NY, USA), and 10% fetal bovine serum (FBS, GIBCO-Life Technologies, Grand Island, NY, USA). After two weeks, MSC reached 90% cell confluence and adherent cells were trypsinized (0.25% Trypsin (Sigma-Aldrich, St. Louis, MO, USA) and 1 mM EDTA). Then, cells were characterized by immunophenotyping and multipotent differentiation capacity assays. MSC were used for all experiments in passages 3-5 to avoid replicative senescence due to prolonged culture conditions.

At the third passage, MSC were trypsinized and stained with specific antibodies for their immunophenotypic characterization: fluorescein isothiocyanate (FITC) mouse anti-human CD73 (clone AD2, BD Pharmingen, San Jose, CA, USA), allophycocyanin (APC) mouse anti-human CD105 (clone SN6, Invitrogen, Frederick, MD, USA), FITC mouse anti-human CD90 (clone F15-42-1, Abcam, Cambridge, MA, USA), and FITC anti-human CD44 (clone MEM-85, Invitrogen, Frederick, MD, USA). Absence of hematopoietic markers was also evaluated with leucocyte-specific antibody PerCP mouse anti-human CD45 (clone 2D1, BD Biosciences, San Jose, CA, USA) and the APC mouse anti-human CD34 (clone 581, BD Pharmingen, San Jose, CA, USA). Data were acquired using a FACSAria II flow cytometer (Becton Dickinson Biosciences, San Jose, CA, USA). FACSDiva software and FlowJo (Becton Dickinson Biosciences, Sunnyvale, CA, USA) were used for data analysis. For the different experiments, immunophenotypic evaluation of MSC in monocultures and MSC in the LN was determined in the same conditions described above.

MSC were cultured in a 24-well plate in IMDM until cell confluence was reached. The MSC multilineage differentiation potential was determined using specific induction protocols and staining for cell type identification by optical microscopy, as follows. For osteogenic differentiation induction, MSC cells were cultured for two weeks with MEM*α* supplemented with 10% FBS, 100 nM dexamethasone, 0.2 mM ascorbic-2-phosphate, and 10 mM *β*-glycerophosphate (all reagents were from Sigma-Aldrich, St. Louis, MO, USA). For chondrogenic differentiation induction, cells were cultured with induction medium (MEM*α* and 10 ng/mL TGF*β*-1, Sigma-Aldrich, St. Louis, MO, USA), also for two weeks. Adipogenic differentiation was achieved by culturing with induction media (MEM*α* supplemented with 10% FBS, 1 mM dexamethasone, 0.5 mM isobutylmethylxanthine, 200 *μ*M indomethacin, and 10 *μ*g/mL insulin; all reagents were from Sigma-Aldrich, St. Louis, MO, USA) every three days alternating with maintenance medium (MEM*α*, supplemented with 10% FBS and 10 *μ*g/mL insulin) for two weeks. For staining, cells were washed three times with PBS (1x), followed by fixation with formaldehyde solution 4% (Sigma-Aldrich, St. Louis, MO, USA), and were stained with 0.35% Oil Red O solution (Sigma-Aldrich, St. Louis, MO, USA) or alkaline phosphatase (using an AP staining kit, EMD Millipore Corporation, Billerica, MA, USA), or with 0.1% Safranin O (Sigma-Aldrich, St. Louis, MO, USA). Cells were examined with an inverted microscope (Eclipse Model TS-100, Nikon, Konan, Minato-ku, Tokyo, Japan) and photographed with a PowerShot A460 ZoomBrowser EX software (Canon, Melville, NY, USA).

### 2.2. Establishment of *In Vitro* Leukemic Niches (LN and REH-CM LN)

The REH cell line (acute lymphocytic leukemia non-T, non-B) was obtained from the American Type Culture Collection (ATCC; CRL-8286, Rockville, MD, USA) and was characterized by flow cytometry for the presence of CD44, CD133, CD38, CD45, and CD19 (data not shown). 6 × 10^4^ BM-MSC were seeded in 24-well plates for 24 h after which they have reached about 80% confluence, then they were cocultured for three days with 5 × 10^4^ total REH cells for the establishment of the leukemic niche (LN). After three days, most REH cells were removed by gently pipetting twice only with cold PBS (1x) and afterwards with PBS EDTA 1 mM (1x). In some experiments, discrimination between MSC and REH cells was also possible by flow cytometry analysis or by CFSE labelling of REH cells. For gene expression analysis and WB (see below), cocultures were extensively washed with PBS EDTA 1 mM in order to remove almost all REH cells. This was monitored by light microscopy, and treatment was stopped when MSC cells were beginning to detach. In this case, the REH contamination was about 3%. In some experiments, the B-ALL cell line SUP-B15 (ATCC; CRL-1929, Rockville, MD, USA) was also used to establish the LN. In parallel, 3 × 10^4^ MSC were seeded in monocultures at less confluence (50%) for three days in IMDM supplemented with 10% FBS as a treatment control. In other experiments, MSC were cultured with fresh REH-conditioned media (see below) for three days to set the REH-CM LN. In this case, cells were re-fed once with fresh REH-CM at half the incubation time.

### 2.3. REH-Conditioned Medium Preparation

2.5 × 10^5^ REH cells/mL were cultured in RPMI 1640 (Invitrogen Corporation, Carlsbad, CA, USA) supplemented with 1% sodium pyruvate, 1% MEM nonessential amino acid solution 100x, and 1% FBS for 24 h at 37°C and 5% CO_2_. Next, REH cells were centrifuged at 500 × *g* for 7 min and the medium was collected and filtered through a 0.22 *μ*m pore membrane filter (Corning Incorporated Pittston, Pittston, PA, USA). The conditioned medium was used fresh in all experiments.

### 2.4. Senescence-Associated *β*-Galactosidase (SA-*β*-Gal) Evaluation in MSC

MSC were cultured as previously described (LN and REH CM-LN or SUP-B15 LN) in duplicates in 24-well plates for three days. For staining, the Cellular Senescence Assay Kit (KA002 Millipore) was used. Next, MSC were washed in the same conditions described above to remove the majority of REH cells from the culture and fixed with formalin 4% for 10 min at room temperature. MSC were washed twice with PBS 1x and incubated at 37°C with *β*-gal substrate overnight in acidic conditions (pH = 6). Positive stained cells were observed and photographed in an inverted microscope (Nikon, Model TS-100, Canon PowerShot A460, ZoomBrowser EX software); the appearance of a perinuclear blue colour was an indication of senescence. Quantification of SA-*β*-Gal activity was performed by manual counting of acquired images (20x magnification) with ImageJ® software (National Institute of Health, USA).

### 2.5. Gene Expression Analysis by qRT-PCR

Monocultured and cocultured (LN and REH CM-LN or SUP-B15 LN) MSC were washed extensively with PBS twice and PBS-EDTA (see above), and total RNA was extracted with TRIzol reagent (Invitrogen). The RNA obtained was quantified by spectrophotometry (NanoDrop 2000C, Thermo Scientific) and stored at -70°C until used. Any possible contamination with genomic DNA was removed using the DNAse I Kit (Invitrogen). Reverse transcription was performed using the High-Capacity Kit (Applied Biosystems, Foster City, CA, USA) from 1000 ng RNA. qRT-PCR was performed on a 7500 real-time PCR Systems (Applied Biosystems®) with the SYBR Green Master Mix (Applied Biosystems, Foster City, CA, USA). Each sample was analysed by triplicate in three independent experiments. The total volume reaction was 20 *μ*L (including 0.5 *μ*L cDNA, SYBR Green, and 0.1 mM of primers). Relative gene expression levels were normalized to GAPDH transcript levels and calculated using the 2^−∆∆CT^ method. To discriminate possible amplifications from REH or SUP-B15 cell lines RNA in LN conditions, we also evaluated the expression of target genes in this cell line.

### 2.6. ROS Level Determination in MSC by Flow Cytometry

MSC cultured in the different conditions were trypsinized and washed twice with PBS 1x. For cytosolic ROS production, cells were incubated with H2-DCFDA 5 *μ*M (Life Technologies®, Thermo Fisher Scientific, Waltham, MA USA) in PBS 1x for 10 min at 37°C; afterwards, cells were washed three times with PBS 1x and resuspended in PBS 1x for FACS analysis. For mitochondrial ROS determination, MSC were incubated with MitoSOX Red® 5 *μ*M (Life Technologies®, Thermo Fisher Scientific, Waltham, MA USA) in PBS 1x 1% BSA for 20 min at 37°C, washed three times with PBS 1x, and resuspended in PBS 1x for FACS analysis. Differentiation between MSC and REH or SUP-B15 cells was possible by using their different forward-scatter and side-scatter signals. Dead cells were excluded during acquisition and analysis (gate: intermediate forward-scatter and low side-scatter). FlowJo (v10.0, FlowJo, LLC, Ashland, OR, USA) was used for data analysis.

### 2.7. Cell Cycle Analysis of MSC in LN Conditions

MSC were trypsinized and washed twice with PBS 1x. Next, cells were fixed with ethanol 70% for 1 h at 4°C and washed three times with PBS 1x. Fixed MSC were incubated with propidium iodide solution (1 mg/mL) and RNAse (1 mg/mL) for 1 h at room temperature. Finally, the supernatants were removed and cells were resuspended in PBS 1x for flow cytometry evaluation in PE-Texas Red channel (FACSAria™ II, BD Biosciences). FlowJo and FCS Express Flow Cytometry Data Analysis Software v5.0 (BD Biosciences) were used for data analysis.

### 2.8. Expression of Adhesion Molecules in MSC and REH Cells after Coculture

Cells were harvested by repeated pipetting after three days, washed in PBS (1x), and stained with monoclonal antibodies for FACS analysis. MSC from the different culture conditions were trypsinized, washed twice with PBS 1x, and stained with different monoclonal antibodies for flow cytometry analysis: PE-conjugated mouse anti-human CD49e (clone IIA1, BD Pharmingen, San Jose, CA, USA), APC-conjugated mouse anti-human CD49d (clone 9F10, BD Pharmingen, San Jose, CA, USA), APC-conjugated mouse anti-human CD54 (clone REA266, Miltenyi Biotec, Auburn, CA, USA), PE-conjugated CD106 (VCAM-1) (clone REA269, Miltenyi Biotec, Auburn, CA, USA), FITC-conjugated mouse anti-human CD44 (clone MEM-85, Invitrogen, Frederick, MD, USA), and PE-conjugated CD184 (CXCR4) (clone 12G5, Miltenyi Biotec, Auburn, CA, USA). The differences between forward-scatter and side-scatter signals in both cell types allowed reliable identification of each. Dead cells were excluded during acquisition and analysis (gate: intermediate forward-scatter and low side-scatter). FlowJo (v10.0, FlowJo, LLC, Ashland, OR, USA) was used for data analysis.

### 2.9. Intracellular SDF-1 Detection in MSC

MSC were fixed for 10 min with 4% formalin in PBS 1x (Sigma). Cells were washed twice with PBS 1x, then permeabilized with Triton X-100 for 10 min at room temperature, and washed again with PBS 1x. Cells were incubated with blocking anti-Fc antibody for 10 min at 4°C. Cells were stained with fluorescein isothiocyanate (FITC) mouse anti-human SDF-1/CXCL12 (clone 79018, Thermo Fisher, Rockford, IL, USA), for 15 min at 4°C. Finally, cells were washed two times with PBS 1x and evaluated for the expression of intracellular SDF-1 by flow cytometry.

### 2.10. Sphere Formation Assay of BM MSC in the LN

For mesensphere formation assay, BM MSC were first labelled with 5 *μ*M carboxyfluorescein diacetate succinimidyl ester (CFSE; CellTrace™ CFSE Cell Proliferation Kit, Invitrogen, Eugene, OR, USA) in PBS 1x supplemented with 0.1% bovine serum albumin (BSA, GIBCO-Invitrogen, Gran Island, NY, USA) for 10 min at 37°C. CFSE-labelled cells were resuspended in IMDM medium (Invitrogen) supplemented with 10% FBS for 5 min on ice and washed three times at 500 × *g* for 5 min at 20°C with IMDM medium, then resuspended in IMDM 10% FBS, and further incubated for 30 min at 37°C to remove the excess of CFSE.

CFSE-labelled MSC were maintained in monocultures for three days in IMDM supplemented with 10% FBS as a control. In parallel, CFSE-labelled MSC were cocultured for three days with REH cells for the establishment of the leukemic niche (LN), in the same conditions described above. To detach REH cells from the MSC, cultures were gently washed twice with cold PBS 1x and then with PBS EDTA 1 mM for 1.5 min. CFSE-labelled MSC (with few remaining REH cells) were centrifuged twice at 500 × *g* for 5 min to remove REH cells. Finally, MSC were counted and plated at low density (15,000 cells/well) in ultralow-adherence 35 mm dishes (Stem Cell Technologies). The growth medium for sphere formation contained 2% B27 supplements (Invitrogen); 20 ng/mL recombinant human basic fibroblast growth factor, 20 ng/mL recombinant human epidermal growth factor, and 1% methylcellulose in Dulbecco's modified Eagle's medium (DMEM)/F12 (1 : 1)/human endothelial (1 : 2) serum-free medium (Invitrogen). As a control, the growth medium without inducers and with methylcellulose was used. The cultures were kept at 37°C with 5% CO_2_ in a water-jacketed incubator and manipulation was minimal to prevent cell aggregation. Afterward, half-medium changes were performed every 48 h for five days. Mesenspheres were observed, measured, counted, and photographed during the five days. Images were acquired using an Eclipse Model TS-100, Nikon, Konan, Minato-ku, Tokyo, Japan, inverted microscope and an Axiovert 40 CFL Zeiss fluorescence microscope.

### 2.11. *In Vitro* Differentiation into Osteogenic, Adipogenic, and Chondrogenic Lineages of MSC after Coculture

Osteo-, adipo-, and chondrogenic differentiation assays of MSC in monocultures and in MSC from the LN, after REH cell removal, were performed as described above. Briefly, 1 × 10^3^ MSC/well were cultured in triplicates in 24-well plates for three days, after which the culture medium was removed and induction media were added and changed periodically as described. Staining and evaluations were done as described above.

### 2.12. REH Cell Proliferation Rate Determined by CFSE Labelling

REH cells previously cocultured with MSC were labelled with 5 *μ*M CFSE (CellTrace™ CFSE Cell Proliferation Kit, Invitrogen, Eugene, OR, USA) in 0.1% BSA (GIBCO-Invitrogen, Grand Island, NY, USA) in PBS 1x for 10 min at 37°C. CFSE-labelled REH cells were suspended in RPMI 1640 medium (Invitrogen Corporation, Carlsbad, CA, USA) with 10% FBS and were let to stand on ice for 5 min and then washed three times at 400 × *g* for 7 min at 20°C with PBS (1x); cells were resuspended in RPMI 1640 with 10% FBS and further incubated 20 min at 37°C. As a negative control of nonproliferating cells (the highest CFSE mean fluorescence intensity), an aliquot of REH cells was FBS-deprived for 48 h (synchronized cells).

### 2.13. REH Cell Migration Capacity Assay

Migration assays of REH cells were performed in transwell chambers (Corning Costar, Tewksbury, MA, USA), with 6.5 mm diameter and 5 *μ*m pore size. Inserts were incubated for 1 h at 37°C with the migration buffer (RPMI 1640/2% BSA). REH cells in monoculture and REH cells previously cocultured with MSC (1 × 10^5^) were added to the upper chambers. Lower chambers had 600 *μ*L of migration buffer. For chemotaxis assays, 100 ng/mL of human recombinant stromal cell-derived factor-1 (hrSDF-1a, Miltenyi Biotec, Auburn, CA, USA) was added to the lower chamber. After 24 h at 37°C and 5% CO_2_ incubation, cells that migrated to the lower chamber were collected and counted in a FACSAria II flow cytometer (BD Biosciences, San Jose, CA, USA). The percentage of migration was calculated by dividing the lower chamber cell number by the total input cell number × 100.

### 2.14. Western Blotting Analysis in MSC

1 × 10^6^ control MSC and MSC from the LN extensively washed to remove the majority of REH cells were lysed with 40 *μ*L of protein extraction buffer (25 mM HEPES (pH 7.7), 0.3 M NaCl, 1.5 mM MgCl_2_, 0.2 mM EDTA, 0.1% Triton X-100, 20 mM *β*-glycerophosphate, and 0.1 mM Na_3_VO_4_) supplemented with protease inhibitors (1 mM phenylmethylsulfonyl fluoride, 20 *μ*g/mL aprotinin, and 20 *μ*g/mL leupeptin). 50 *μ*g of proteins was separated in SDS polyacrylamide gels, transferred to PVDF filters, blocked with 5% (*w*/*v*) power defatted milk in TBS-T (50 mM Tris-HCl (pH 8.0), 150 mM NaCl, and 0.1% Tween-20) for 1 h at room temperature, and incubated overnight at 4°C with the following antibodies: phospho-NF-*κ*B p65 (Ser536) monoclonal antibody (Abcam, Cambridge, MA, USA) and total NF-*κ*B p65 monoclonal antibody (Abcam, Cambridge, MA, USA). Signals were developed by colorimetric methods using an NBT/BCIP Colour Development Substrate Kit (Promega, Madison, WI, USA).

### 2.15. Adhesion Kinetics of Leukemic Cell Lines to MSC

To evaluate the adhesion of leukemic cells to MSC at different time points (0, 1, 2, 4, and 6 h), 6 × 10^4^ MSC were seeded in 24-well plates in triplicates. MSC were cocultured with 5 × 10^4^ REH or SUP-B15 cells. After the incubation period, supernatants were collected and cell count was performed in a Neubauer chamber. The percentage of leukemic cells adhered to MSC was calculated based on the input of the leukemic cells.

### 2.16. Population Doubling Time of REH and SUP-15 Cells

To evaluate the cellular growth of REH and SUP-B15 cells, they were seeded in duplicates at two cell densities (5 × 10^3^ and 1 × 10^4^ cells/well). Cells were collected and counted at 6 and 12 h and 1, 2, 3, 4, 5, 6, and 7 days.

### 2.17. Statistical Analysis

SA-*β*-Gal activity, ROS production, cell cycle, REH cell proliferation, and migration data were analysed using Student *t*-test (unpaired *t*-test). SASP quantification, adhesion molecule expression, gene expression, and formation of spheres and adhesion kinetics were analysed with Kruskal-Wallis test (nonparametric one-way ANOVA). The medium comparison was made with Dunn's multiple comparison test. Statistical tests were used for nonparametric data. GraphPad Prism 5.0 (GraphPad Software Inc., La Jolla, CA, USA) was used for mathematical calculations and graphics. Results were considered significant when *p* < 0.05.

## 3. Results

### 3.1. MSC in the LN Showed Senescence-Associated *β*-Galactosidase Activity and Increased p53 Expression

**Figure 1:**
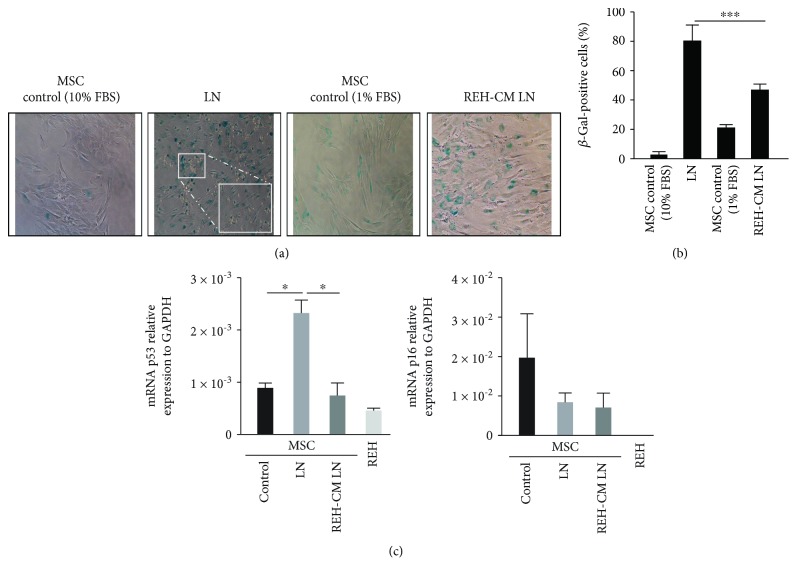
Leukemic cells increase senescence-associated *β*-galactosidase (SA-*β*-Gal) activity in MSC. (a) SA-*β*-Gal activity in MSC cocultured with REH cells (LN) or REH-CM (REH-CM LN) was measured. As controls, we have used MSC in 10% FBS (for the LN) and 1% FBS (for the REH-CM LN). Inset: magnification of the LN, showing absence of SA-*β*-Gal activity in REH cells. (b) Percentage of positive cells for SA-*β*-Gal activity in the different culture conditions (*p* values: Student *t*-test-Mann-Whitney test; ^∗∗∗^ *p* < 0.001). (c) qRT-PCR quantification of p53 and p16 gene expression in MSC cultured in the LN and REH-CM LN for three days. Results represent three independent experiments done in duplicates. Results are expressed as mean ± SEM (*p* values: nonparametric one-way ANOVA; ns: nonsignificant, ^∗^ *p* < 0.05).

We have previously established an *in vitro* LN model to study HSC functions under leukemic stress [14, 24]. This *in vitro* system showed that HSC are affected in a similar way as HSC and HPC do in leukemic patients; therefore, we envisaged that this LN could also be a useful tool for studying the phenotypic and functional alterations of MSC during leukemic cell growth. BM MSC (Supplementary Figures 1(a)–1(c)) were cultured with REH cells for three days (establishing the LN), and afterwards, MSC evaluation was performed. We have previously demonstrated that three days of coculture of MSC with REH cells are sufficient to detect HSC phenotypic and functional alterations [14]; therefore, we assumed that three days would also be appropriate to evaluate MSC in the LN. Previously, we have also shown that incubation of MSC with a REH-conditioned medium (REH-CM) for three days induced a flattened morphology in MSC with the appearance of cytoplasmic vacuoles and, importantly, cytoplasmic senescence-associated *β*-galactosidase (SA-*β*-Gal) activity [24]. As expected, the coculture of REH cells with MSC also induced SA-*β*-Gal activity in approximately 80% of the MSC while we observed only 5% of positive cells in control MSC (Figures 1(a) and 1(b)). The increase in the number of SA-*β*-Gal activity-positive cells was also observed, although to a lesser extent (50%), when the LN was established with the REH-CM (REH-CM LN) (Figures 1(a) and 1(b)), suggesting that both direct cell-to-cell contacts and soluble factors have a role in SA-*β*-Gal induction. We also found that MSC in the LN exhibited higher (almost threefold increase) p53 gene expression compared to control MSC or REH-CM LN (Figure 1(c)), implying that direct cell-to-cell contact has a greater effect on the mechanism of induction of senescence. This could be due to the fact that cytokine release and the corresponding effect on the neighbouring cells is more efficient in cells that are bound to each other. On the other hand, REH cells showed a lower expression of p53 (Figure 1(c)), and as observed in the remaining cells in the coculture, they do not stain for SA-*β*-Gal (Figure 1(a), inset in LN). No statistically significant differences were observed in p16 expression in the different settings (Figure 1(c), right panel). REH cells were negative for the expression of p16.

### 3.2. Oxidative Stress Has a Major Role in MSC Senescence

**Figure 2:**
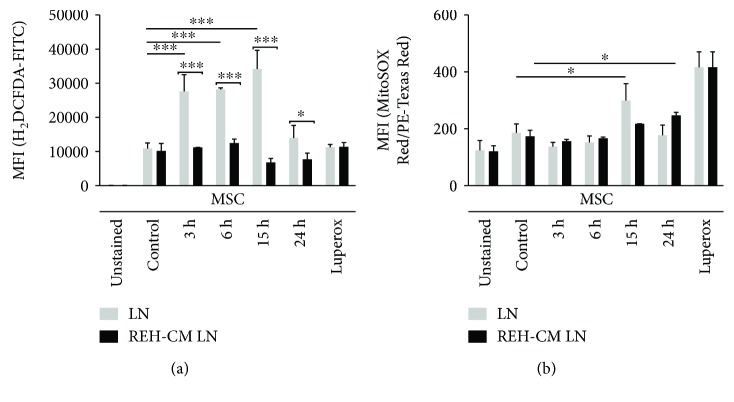
ROS induction in MSC under leukemic conditions. (a) Cytosolic (H_2_-DCFDA) and (b) Mitochondrial (MitoSOX Red™) ROS were measured in MSC cultured in LN and REH-CM LN at the different time points indicated. MSC ROS quantification was made by flow cytometry analysis. Luperox® treatment (200 *μ*M for 2 h) was used as a positive control of acute oxidative stress. Results represent three independent experiments done in duplicates (*p* values: Student *t*-tests; ns: nonsignificant, ^∗^ *p* < 0.05, ^∗∗∗^ *p* < 0.001).

An important mechanism of cellular senescence induction is the production of reactive oxygen species (ROS). Oxidative stress can produce irreversible DNA damage and consequently cell cycle arrest. We have measured MSC ROS induction both in LN and in REH-CM LN. As a positive control, MSC were treated with tert-butyl hydroperoxide (Luperox®), which acts as a potent oxidative inducer. A reliable identification of ROS production in the MSC was possible by their different forward-scatter and side-scatter signals compared to REH cells. No ROS production was detected in REH cells. ROS production was measured in MSC during the first 3 to 24 h. First, MSC cytosolic ROS species production (H2-DCFDA staining) was evident after 3 h of coculture, and it was persistent for an additional 12 h (Figure 2(a), LN). ROS levels returned to basal values after 24 h. Cytoplasmic ROS assessment in MSC analysed from the REH-CM LN showed no important changes during the period of time evaluated (Figure 2(a), REH-CM LN). Mitochondrial ROS production by MSC (MitoSOX Red®) was only significant after 15 h of coculture in the LN (Figure 2(b), LN) and after 24 h in the REH-CM LN (Figure 2(b), REH-CM LN). These results showed that senescence in MSC was mediated by oxidative stress induction and that direct contact with REH cells seems to play a major role or is more efficient than just incubation with the REH-CM.

### 3.3. Leukemic Cells Produce a G2/M Phase Arrest of the Cell Cycle in MSC

**Figure 3:**
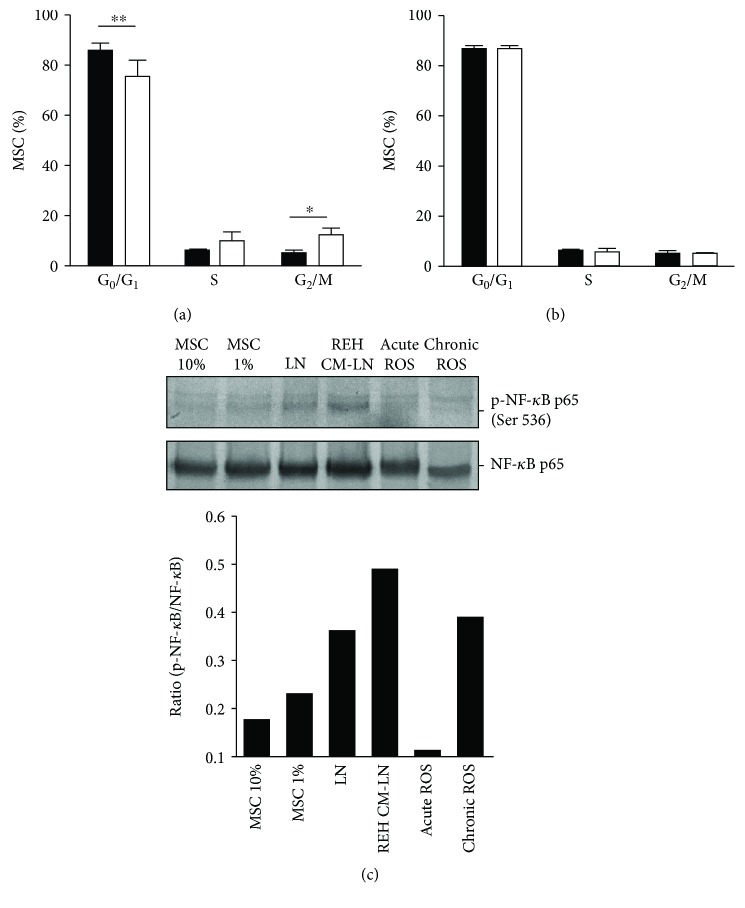
Cell cycle analysis and NF-*κ*B activation in MSC under leukemic conditions. (a) MSC were cocultured with REH cells for three days. Cell cycle evaluation was performed by propidium iodide staining and flow cytometry analysis, comparing MSC in monoculture (black bars) and MSC cultured in LN (white bars). (b) MSC were cultured with REH-CM for three days. Results are expressed as percentages of MSC in each phase of the cell cycle of three independent experiments done in duplicates (*p* values: Student *t*-tests; ns: nonsignificant, ^∗^ *p* < 0.05 and ^∗∗^ *p* < 0.01). (c) NF-*κ*B p65 phosphorylation at Ser536 in MSC at different conditions of culture (upper panel) and quantification by densitometry in relation to NF-*κ*B protein expression (lower panel).

Permanent cell cycle arrest is a typical sign of senescent cells. We observed that MSC in monoculture remain in a quiescent state (86% of cells in the G1/G0 phase), while MSC cocultured with REH cells showed a decrease in the G1/G0 phase (75% of cells) and a significant increase of cells in the G2/M phase (4% of cells in MSC monoculture vs. 11% of cells in LN conditions) (Figure 3(a)). These changes were not observed in MSC from the REH-CM LN (Figure 3(b)).

### 3.4. NF-*κ*B Activation in MSC from the LN

We also evaluated NF-*κ*B activation by phosphorylation of the p65 subunit at the Ser536 residue. In these experiments, the cocultures were washed extensively with PBS-EDTA to remove almost all REH cells, as explained above in Materials and Methods. We found that in both leukemic conditions (LN and REH CM-LN), there was an activation of this signalling pathway, probably mediating the induction and maintenance of senescence in MSC (Figure 3(c)). These results showed the important role of the NF-*κ*B pathway in MSC senescence induction under leukemic conditions in our *in vitro* model.

### 3.5. MSC Adhesion Molecule Expression Is Altered in the LN

**Figure 4:**
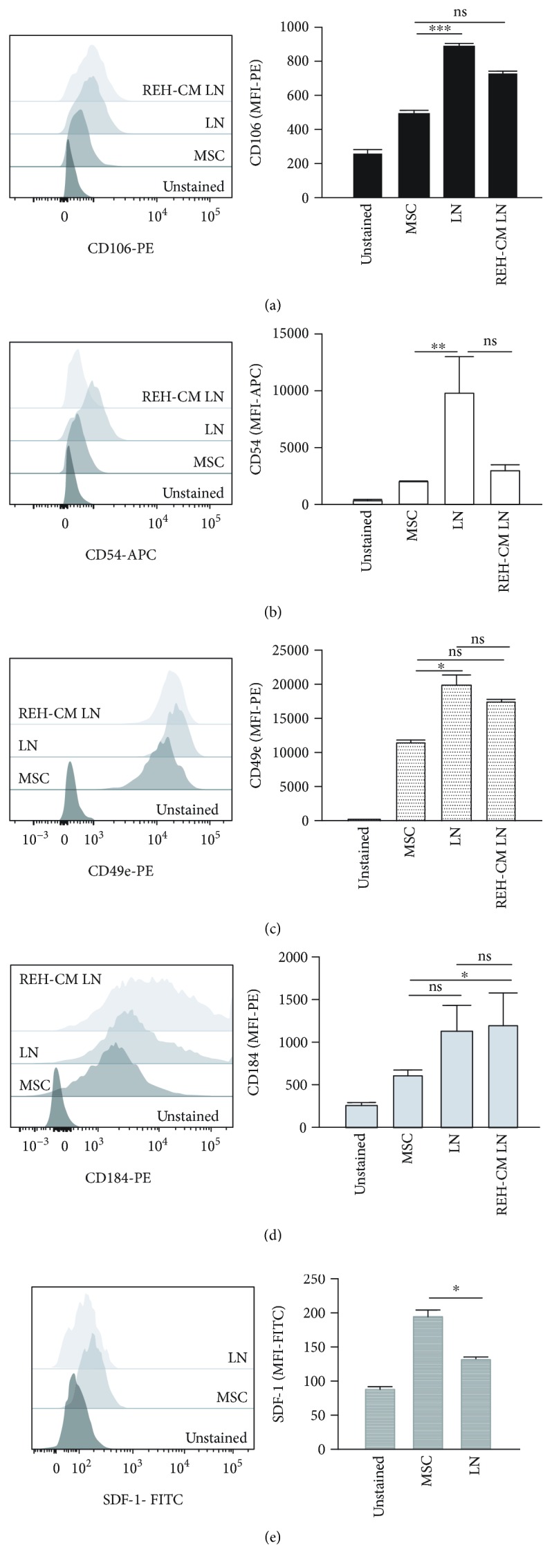
Adhesion molecule expression in MSC from the LN and the REH-CM LN. MSC were cocultured with REH cells and REH-CM for three days. Flow cytometry analysis of (a) CD106, (b) CD54, (c) CD49e, (d) CD184, and (e) SDF-1 were measured as indicated. Results are expressed as the median fluorescence intensity (MFI) from two independent experiments done in triplicates (*p* values: Student *t*-tests; ns: nonsignificant, ^∗^ *p* < 0.05, ^∗∗^ *p* < 0.01, and ^∗∗∗^ *p* < 0.001).

During the course of the experiments, we noticed that REH cells seem to strongly adhere to MSC. We then performed an adherence functional assay and showed that REH cells adherence to MSC increases over time (Supplementary Figure 2(b)). Next, we evaluated the expression of different adhesion molecules in MSC which could be relevant in the interaction with the leukemic cells. VCAM-1 (CD106) expression in MSC showed an increase in LN (1.5-fold increase) (Figure 4(a)). Likewise, MSC showed a remarkable increase in ICAM-1 (CD54) expression in the LN, which was not observed in the REH-CM LN (Figure 4(b)). MSC CD49e expression increased in both conditions, LN and REH-CM LN (Figure 4(c)). CXCR4 (CD184) expression was more variable in MSC but was also increased in both LN and REH-CM LN (Figure 4(d)). This increase in adhesion molecule expression may explain the higher adhesion of REH cells to MSC observed previously in the LN [14], but the real contribution of each molecule to this adherence will require a careful analysis with the use of specific antibodies against the inactive and active forms of the adhesion molecules. Interestingly, SDF1 expression was reduced in MSC from the LN (Figure 4(e)).

### 3.6. Impaired MSC Stemness Function in the LN

**Figure 5:**
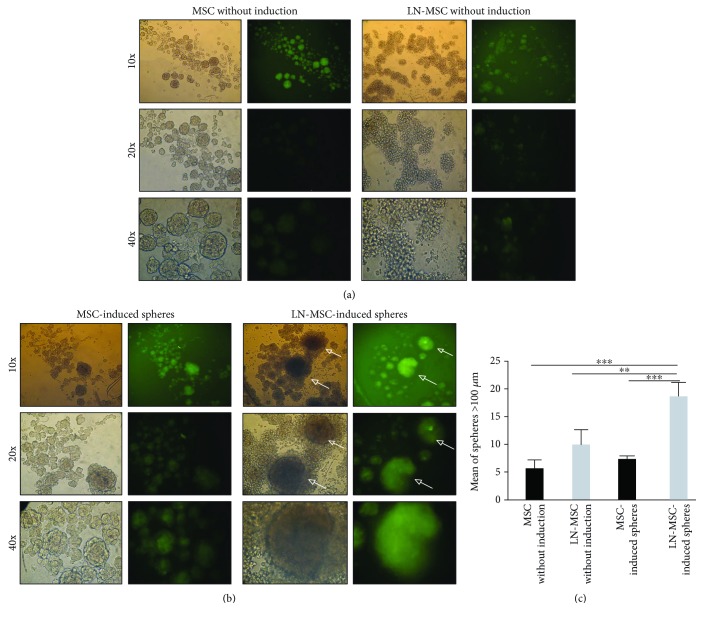
Human MSC formed mesenspheres after coculture with REH cells. MSC and LN-MSC spheres formed (a) without or (b) with induction. MSC or LN-MSC were previously labelled with CFSE. Representative photographs of mesenspheres are shown at different magnifications (10x, 20x, and 40x). (c) Mean number of spheres with a diameter > 100 *μ*m in the different culture conditions. Results are expressed as mean ± SEM (*p* values: nonparametric one-way ANOVA; ^∗∗^ *p* < 0.01 and ^∗∗∗^ *p* < 0.001).

Murine and human MSC can form clonal mesenspheres with the ability to self-renew and differentiate into mesenchymal lineages. We have therefore explored if leukemic stress could affect these stemness cell properties in MSC. Induction of mesensphere formation in MSC produced spheres with a well-defined morphology and of different sizes (Supplementary Figure 1(c)). Interestingly, MSC have a basal (without induction) capacity to form spheres (Figure 5(a), left panel); on the contrary, MSC obtained from the LN form irregular and smaller mesenspheres under basal conditions (Figure 5(a), right panel). Induced MSC spheres in the LN were more and larger than in normal condition (Figure 5(b), right panel, and Figure 5(c)). CFSE labelling of MSC allowed the clear demonstration that mesenspheres were formed by MSC and not by REH cells, the latter seen surrounding completely the mesenspheres (Figure 5(b), arrows). Of note, in spite of having removed the majority of REH cells, they grew abundantly and were seen in contact with mesenspheres.

**Figure 6:**
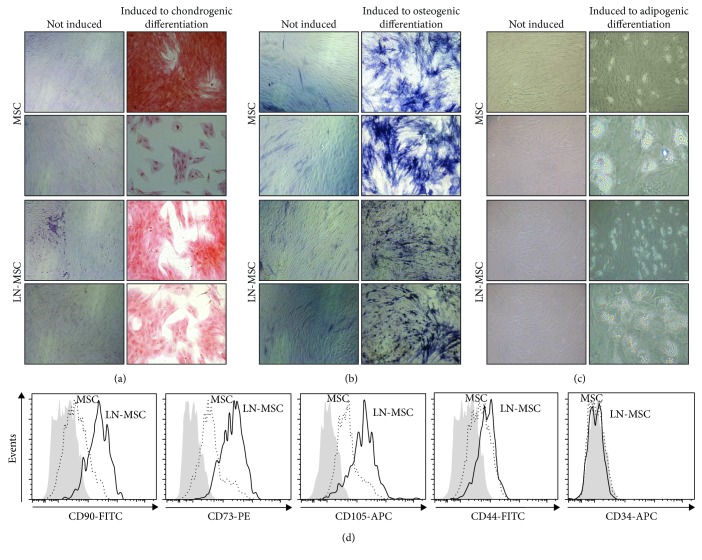
In vitro multipotent differentiation capacity and phenotypic characterization of MSC after coculture with REH cells. (a) MSC differentiation capacity to chondrocytes in monoculture (MSC) and coculture with REH cells (LN-MSC) for three days detected by Safranin O staining (photographs magnification 10x). (b) MSC differentiation capacity of MSC to osteoblasts in monoculture and coculture with REH cells for three days detected by ALP staining (photographs magnification 10x). (c) MSC differentiation capacity of MSC to adipocytes in monoculture and coculture with REH cells for three days. (d) MSC LN immunophenotypic characterization by flow cytometry (dark grey histograms: isotype controls; antigen expression in MSC (dotted line) and MSC LN (solid line), as indicated in the figure).

The multilineage differentiation capacity of MSC cultured with leukemic cells was partially affected (Figure 6). Whereas chondrogenic and adipogenic differentiation were similar in MSC obtained from control cultures or LN (Figures 6(a) and 6(c)), a reduced ability to differentiate to the osteoblastic lineage, as evaluated by a more diffuse and faint ALP staining, was observed (Figure 6(b)). This was somehow unexpected since surface marker evaluation of MSC, in coculture with REH cells, consistently showed higher expression of CD105 (endoglin, the TGF-beta receptor III), a marker present in osteoprogenitors [25] (Figure 6(d)). The expression of other markers (CD90, CD73, and CD44) was also increased (Figure 6(d)), but their variation was more inconsistent in the different experiments. MSC remained negative for CD34 and CD45 during LN cultures (Figure 6(d) and not shown). Altogether, these results showed that MSC stemness is partially compromised in the LN.

### 3.7. REH Cells Showed Higher Proliferation and Migration Capacity after Incubation in the LN

**Figure 7:**
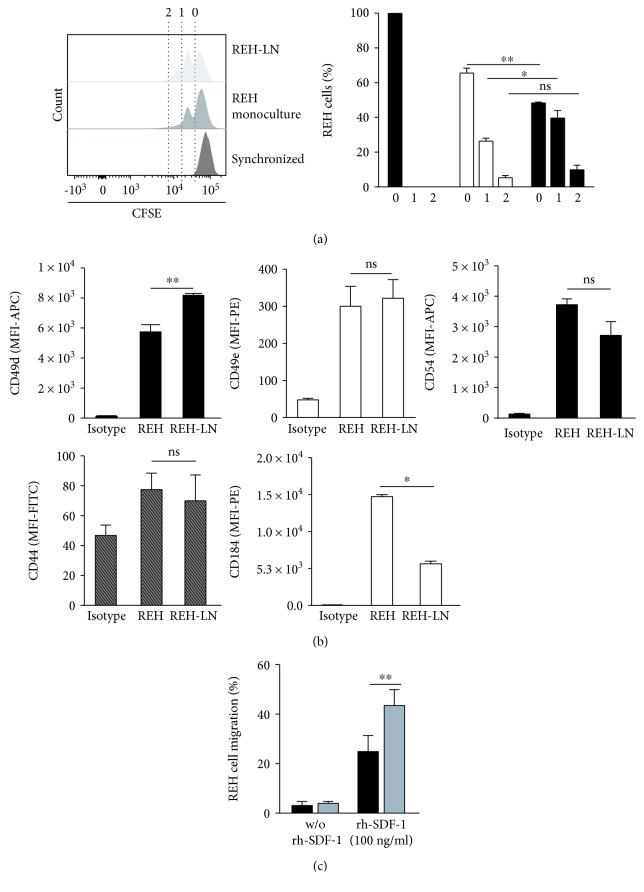
REH cell evaluation after MSC coculture. (a) REH cells were cocultured with MSC for three days after which cell proliferation was assessed. Proliferation capacity was measured by cell staining with CFSE (left panel). Cells were synchronized by serum starvation (FBS removal for two days). Percentage of synchronized REH cells (black bars), monoculture (white bars), and coculture (grey bars) at the corresponding number of cell division (0, 1, and 2) are shown (right panel). (b) Adhesion molecule expression in REH cells cultured in LN conditions for three days. Median fluorescence intensity (MFI) of CD49d, CD49e, CD54, CD44, and CD184 is shown. (c) REH cell migration capacity towards SDF-1 (100 ng/mL) was determined in a transwell insert with a 5 *μ*m pore membrane. REH cells in monoculture and coculture with MSC were allowed to migrate for 24 h, after which cells in the lower chamber were harvested and counted by flow cytometry. The percentage of migration was calculated considering the total input of REH cells. Data were obtained from two independent experiments done in triplicates (*p* values: Student *t*-test; ns: nonsignificant, ^∗^ *p* < 0.05 and ^∗∗^ *p* < 0.01).

We have also evaluated some REH cell properties in the LN. We found that REH cells increased cell proliferation when cocultured with MSC (Figure 7(a)). 40% of the REH cells in coculture have completed the first cell division, compared to only 25% in monoculture (Figure 7(a), right panel). Except for an increase in CD49d, the expression of the adhesion molecules CD49e, CD54, and CD44 in REH cells was similar in the LN and in monoculture (Figure 7(b)). CXCR4 (CD184) expression in REH cells in LN was importantly reduced compared to control cells (Figure 7(b)). Finally, we found an important increase in migration capacity of REH cells obtained from the LN towards the chemoattractant SDF-1 (100 ng/mL) (Figure 7(c)). Nondirected migration without SDF-1 was minimal and very similar to control cells. These results showed that REH cells have a higher cell proliferation capacity and SDF-1-directed migration competence in the LN.

### 3.8. Another Leukemic Cell Line Induced Similar Changes in MSC from the LN

To explore if another leukemic cell line would produce equivalent changes in the MSC, we have also established a LN by coculturing MSC with the B-ALL cell line SUP-B15. Although the proliferation rate of this cell line without MSC was reduced compared to REH cells (Supplementary Figure 2(a)), adherence to MSC was equivalent (Supplementary Figure 2(b)). As expected, SA-*β*-Gal activity was increased in MSC from this LN (Supplementary Figures 3(a) and 3(b)). In this case, we found that the mediator of the senescence process was p16, instead of p53 as found in the REH cell line (Supplementary Figure 3(d)). Nevertheless, cytoplasmic and mitochondrial ROS were increased in MSC from the LN established with SUP-B15, similar to the LN with the REH cell line, although some small differences were observed (Supplementary Figures 4(a) and 4(b)). The reason for these variations needs further experimentation, but they may be due, first and obviously, to intrinsic differences between the two cell lines. Nevertheless, these results suggest that MSC are affected in a similar manner by different leukemic cells. Evaluation of primary cultures from B-ALL patients' samples will show the relevance of these findings and their possible practical usefulness.

## 4. Discussion

It has been shown that leukemic cells induce considerable modifications in the bone marrow microenvironment with considerable effects over its architecture and the different cell populations present. These changes are not only permissive to leukemic cell growth but also favour resistance to chemotherapeutic treatments and leukemic relapse [16, 26]. Here, we used an *in vitro* model of LN [14] to evaluate the functional and molecular changes that occur in MSC and how these niche alterations endow REH cells with new capabilities.

We found that direct contact of leukemic cells with MSC induced a senescence process in MSC (as evaluated by SA-*β*-Gal induction) that could only be partially (40-50%) reproduced by the soluble factors from a REH-conditioned medium. Activation of the p53/p21 or pRb/p16 signalling pathways has been connected with persistent stress and cell cycle arrest in aging cells [22, 27–29]. We observed an increased expression of p53 in MSC cultured in LN conditions but not in REH CM-LN, suggesting that stress induction by cell-to-cell contact with REH cells is stronger than by soluble factors. This could be due to the fact that release and effects of cytokines are more efficient in cells that are attached to each other. Interestingly, MSC obtained from AML patients showed increased SA-*β*-Gal, and it was suggested that it could have been mediated by p53/p21 overexpression [30]. p16 expression was unaffected, consistent with previous reports showing that the activation of one or the other pathway depends on the nature of the stress trigger [31, 32]. Since low and persistent oxidative stress activates the senescence response [23, 33, 34], we have evaluated ROS production in MSC at different time-set points. We found an early and persistent cytoplasmic ROS production in MSC starting at 3 h of culture and persisting until 15 h in the LN. On the other hand, mitochondrial ROS production was only evident after 15 h. As happened with SA-*β*-Gal induction, the REH-CM LN was less effective in ROS induction, with no cytoplasmic detection and modest mitochondrial ROS induction only at 24 h. Of note, when the LN was set with another B-type ALL (SUP-B15), we observed equivalent effects in MSC, i.e., SA-*β*-Gal induction and increase in both p16 expression and cytoplasmic and mitochondrial ROS production. These results demonstrate that MSC senescence under leukemic stress is mediated by ROS induction, due to cell-to-cell contact and soluble factor release from MSC in the presence of abundant leukemic cells.

Cell cycle arrest, the initial sign of cellular senescence, occurring either at G1/G0 (mainly associated with p16 induction) or at G2/M (mainly associated with p53 activation) phases [23, 35, 36], was also evaluated. A slightly but significant increase in the number of cells in the G2/M phase in MSC from the LN was observed. An early damage of cells in the G2 phase of the cell cycle with a rapid activation of the p53/p21 pathway would induce cell cycle arrest in our *in vitro* system [36, 37]. This was not observed in MSC incubated with the REH-CM, suggesting that cell-to-cell interactions play a major role or are more efficient in cytokine release and in the observed effects.

Senescent cells are characterized by the production of the so-called senescence-associated secretory phenotype (or SASP), a particular set of chemokines, cytokines, and growth factors. Previously, we have identified that the main secreted factors in both LN conditions (LN and REH CM-LN) were IL-6, IL-8, and CCL2 [14, 38], a modified MSC secretion profile dependent on p53 expression [37] and apparently protecting leukemic cells [39]. In our *in vitro* model, early senescence induction has been mediated by p53 expression and later could have been reinforced by SASP production, as in other cancer models [40].

Of note, SASP secretion produces substantial changes in MSC adhesion molecule expression such as VCAM-1 and ICAM-1, among others [41, 42]. We observed an important increase in VCAM-1 (CD106), ICAM-1 (CD54), and VLA5 (CD49e) expression in the LN, and to a lower extent in the REH-CM LN. Although a direct demonstration is missing, these molecules could be involved in the high adherence between MSC and REH cells observed before [14] and here. The interaction of MSC VCAM1 with leukemic cells VLA4 has been shown to be relevant for chemotherapy resistance in AML and ALL by a NF-*κ*B-mediated mechanism [43, 44]. We have also shown here that VLA5 (CD49e) is upregulated in REH cells after interactions with MSC, suggesting that in our system VCAM-1/VLA5 interaction and signalling could be relevant for conferring advantageous properties to leukemic cells. As a proof of this, we found NF-*κ*B activation in MSC in the LN and the REH-CM LN.

According to this, REH cells showed a significant increase in their proliferation capacity after MSC contact, as it has been previously described in other ALL cell lines and ALL patient leukemic cells [16, 45, 46]. In our *in vitro* model, leukemic cell proliferation could be further fostered by the selected SASP induced during the senescence process. Furthermore, REH cell migration capacity was increased in LN culture conditions. This may be the result of the joint action of a decrease in SDF1 production in MSC and a reduction in CXCR4 expression in REH cells, as shown here in our systems, conditions that *in vivo* could favour the colonization of new proximal niche [47].

Importantly, MSC stemness functions were partially altered. First, mesensphere formation was dramatically increased in the LN. The real significance of this is unknown, but it could mean that MSC are augmented under these LN conditions or that these MSC acquired particular properties (increased adhesion molecule expression or increased soluble factor secretion) allowing leukemic cells to nest and grow efficiently. In fact, we noticed that in spite the small number of REH cells left in the cultures during the sphere assays, they were able to proliferate avidly and locate abundantly around the spheres. This interesting subject merits further experimentation. On the other hand, MSC in the LN inefficiently differentiate into the osteoblastic lineage, suggesting that abnormal differentiation capacity is a hallmark of these MSC cells in contact with leukemic cells. This has been also noticed in patients with CML or cell line models of CML (17). Also, in AML patients, degradation of osteoblasts and the endosteal endothelium was observed (18). Finally, in paediatric ALL patients, an osteoblast decrease has also been reported [48].

All in all, our results showed that during the growth of the ALL REH or the B-ALL SUP-B15 cell lines, MSC begin a senescent process probably mediated by p53 or p16 and ROS production with a subsequent arrest in the G2/M phase of the cell cycle (REH cells). Direct cell-to-cell contact between MSC and REH cells seems to be the main mechanisms responsible for these changes, probably as a result of efficient delivery of cytokines. In fact, a high expression of specific adhesion molecules was observed in MSC and REH cells in the LN. Importantly, NF-*κ*B activation was observed in MSC in the LN, a signalling pathway that has been linked to high VCAM-1 expression and chemotherapy resistance. Intriguingly, MSC stemness functions were also affected in this LN. Some of the here observed changes have been reported also in MSC from different leukemic patients. We propose our *in vitro* system as a bone fide LN model useful for the elucidation of the different mechanisms involved in BM niche damages produced by the leukemic cell growth, affecting HSC functions and supporting preferentially leukemic cells. This model can also provide relevant information about cellular targets that could block leukemic cell growth or resistance to chemotherapy.

## 5. Conclusions

In this *in vitro* system, leukemic cells induced important modifications in MSC including the induction of a senescence process and impaired multipotent differentiation capacity. Cell-to-cell contact and soluble factors are responsible for the observed changes. In spite of this process taking place, REH cells proliferate more and have augmented directed migration towards SDF-1. This *in vitro* LN could be useful for studying the molecular and cellular mechanisms responsible for these effects and for exploring novel therapeutic targets.

## Data Availability

The data used to support the findings of this study are available from the corresponding author upon request.

## Supplementary Materials

Supplementary Materials These experiments are related to the identification and characterization of MSC and to the effect on MSC of another leukemia cell line (SUP-B15) tested, to show that the results obtained are similar to the ones obtained with the leukemic REH cell line. Supplementary Figure 1: isolation and characterization of MSC. (a) BM-MSC immunophenotypic characterization by flow cytometry (dark grey histograms: isotype controls; antigen expression: light grey histograms). Cell surface markers and fluorochromes used are shown in each histogram. (b) Differentiation potential of BM-MSC; adipogenic differentiation showing lipid-rich vacuoles stained with Oil Red O (magnification 10x); osteogenic differentiation detected by ALP staining (magnification 10x); chondrogenic differentiation observed by Safranin O staining (magnification 10x). Left panels show control cells without induction. (c) Sphere-forming ability of BM MSC. A representative BM-MSC sample is shown. Supplementary Figure 2: cell growth and cell adhesion capacity of the ALL cell lines REH and SUP-B15. (a) The cell growth rate of REH and SUP-B15 cells was evaluated for 7 days at the different time points indicated after thawing fresh vials. (b) Percentage of adhesion of the REH and SUP-B15 cells to the MSC at 0, 1, 2, 4, and 6 h of coculture. Results are expressed as mean ± SEM (*p* values: nonparametric one-way ANOVA; ^∗^ *p* < 0.05 and ^∗∗∗^ *p* < 0.001). Supplementary Figure 3: SUP-B15 cells induce increased SA-*β*-Gal activity in MSC. (a) Representative micrographs (10x) of SA-*β*-Gal activity in MSC control (10% FBS) and MSC cocultured with SUP-B15 cells (SUP-B15 LN). Inset: magnification of the LN, showing absence of SA-*β*-Gal activity in SUP-B15 cells. (b) Quantification (%) of positive cells for SA-*β*-Gal activity in the different culture conditions (Student *t*-test, ^∗∗^ *p* < 0.01). (c) mRNA expression of p53 and p16 in MSC cultured in the SUP-B15 LN for three days. Results represent two independent experiments done in duplicates. Results are expressed as mean ± SEM (*p* values: nonparametric one-way ANOVA; ns: nonsignificant, ^∗^ *p* < 0.05, ^∗∗^ *p* < 0.01, and ^∗∗∗^ *p* < 0.001). Supplementary Figure 4: production of ROS in MSC cocultured with SUP-B15 cells. (a) Mean fluorescence intensity of the cytosolic oxidative stress indicator H2-DCFDA in MSC of SUP-B15-LN and (b) mean fluorescence intensity of MitoSOX Red™ (Mitochondrial ROS). Results are expressed as mean ± SEM (*p* values: nonparametric one-way ANOVA; ^∗^ *p* < 0.05, ^∗∗^ *p* < 0.01, and ^∗∗∗^ *p* < 0.001).

## References

[1] Schofield R. (1978). The relationship between the spleen colony-forming cell and the haemopoietic stem cell. *Blood Cells*.

[2] Lilly A. J., Johnson W. E., Bunce C. M. (2011). The haematopoietic stem cell niche: new insights into the mechanisms regulating haematopoietic stem cell behaviour. *Stem Cells International*.

[3] Boulais P. E., Frenette P. S. (2015). Making sense of hematopoietic stem cell niches. *Blood*.

[4] Morrison S. J., Scadden D. T. (2014). The bone marrow niche for haematopoietic stem cells. *Nature*.

[5] Anthony B. A., Link D. C. (2014). Regulation of hematopoietic stem cells by bone marrow stromal cells. *Trends in Immunology*.

[6] Schraufstatter I. U., Discipio R. G., Khaldoyanidi S. (2011). Mesenchymal stem cells and their microenvironment. *Frontiers in Bioscience*.

[7] Docheva D., Popov C., Mutschler W., Schieker M. (2007). Human mesenchymal stem cells in contact with their environment: surface characteristics and the integrin system. *Journal of Cellular and Molecular Medicine*.

[8] Méndez-Ferrer S., Michurina T. V., Ferraro F. (2010). Mesenchymal and haematopoietic stem cells form a unique bone marrow niche. *Nature*.

[9] Lane S. W., Scadden D. T., Gilliland D. G. (2009). The leukemic stem cell niche: current concepts and therapeutic opportunities. *Blood*.

[10] Lim M., Pang Y., Ma S. (2016). Altered mesenchymal niche cells impede generation of normal hematopoietic progenitor cells in leukemic bone marrow. *Leukemia*.

[11] van den Berk L. C. J., van der Veer A., Willemse M. E. (2014). Disturbed CXCR4/CXCL12 axis in paediatric precursor B-cell acute lymphoblastic leukaemia. *British Journal of Haematology*.

[12] André T., Meuleman N., Stamatopoulos B. (2013). Evidences of early senescence in multiple myeloma bone marrow mesenchymal stromal cells. *PLoS One*.

[13] Nwajei F., Konopleva M. (2013). The bone marrow microenvironment as niche retreats for hematopoietic and leukemic stem cells. *Advances in Hematology*.

[14] Vernot J.-P., Bonilla X., Rodriguez-Pardo V., Vanegas N.-D. (2017). Phenotypic and functional alterations of hematopoietic stem and progenitor cells in an in vitro leukemia-induced microenvironment. *International Journal of Molecular Sciences*.

[15] Riether C., Schürch C. M., Ochsenbein A. F. (2015). Regulation of hematopoietic and leukemic stem cells by the immune system. *Cell Death & Differentiation*.

[16] Zhou H.-S., Carter B. Z., Andreeff M., Zhou H.-S., Carter B. Z., Andreeff M. (2016). Bone marrow niche-mediated survival of leukemia stem cells in acute myeloid leukemia: Yin and Yang. *Cancer Biology & Medicine*.

[17] Kumar A., Anand T., Bhattacharyya J., Sharma A., Jaganathan B. G. (2018). K562 chronic myeloid leukemia cells modify osteogenic differentiation and gene expression of bone marrow stromal cells. *Journal of Cell Communication and Signaling*.

[18] Duarte D., Hawkins E. D., Akinduro O. (2018). Inhibition of endosteal vascular niche remodeling rescues hematopoietic stem cell loss in AML. *Cell Stem Cell*.

[19] Garderet L., Mazurier C., Chapel A. (2007). Mesenchymal stem cell abnormalities in patients with multiple myeloma. *Leukemia & Lymphoma*.

[20] Pérez-Mancera P. A., Young A. R. J., Narita M. (2014). Inside and out: the activities of senescence in cancer. *Nature Reviews Cancer*.

[21] Lasry A., Ben-Neriah Y. (2015). Senescence-associated inflammatory responses: aging and cancer perspectives. *Trends in Immunology*.

[22] Salama R., Sadaie M., Hoare M., Narita M. (2014). Cellular senescence and its effector programs. *Genes & Development*.

[23] Correia-Melo C., Hewitt G., Passos J. F. (2014). Telomeres, oxidative stress and inflammatory factors: partners in cellular senescence?. *Longevity & Healthspan*.

[24] Vanegas N. D. P., Vernot J. P. (2017). Loss of quiescence and self-renewal capacity of hematopoietic stem cell in an in vitro leukemic niche. *Experimental Hematology & Oncology*.

[25] Aslan H., Zilberman Y., Kandel L. (2006). Osteogenic differentiation of noncultured immunoisolated bone marrow-derived CD105^+^ cells. *Stem Cells*.

[26] Tabe Y., Konopleva M. (2014). Advances in understanding the leukaemia microenvironment. *British Journal of Haematology*.

[27] Vigneron A., Vousden K. H. (2010). p53, ROS and senescence in the control of aging. *Aging*.

[28] Rayess H., Wang M. B., Srivatsan E. S. (2012). Cellular senescence and tumor suppressor gene p16. *International Journal of Cancer*.

[29] Qian Y., Chen X. (2013). Senescence regulation by the p53 protein family. *Cell Senescence*.

[30] Kornblau S. M., Ruvolo P. P., Wang R. Y. (2018). Distinct protein signatures of acute myeloid leukemia bone marrow-derived stromal cells are prognostic for patient survival. *Haematologica*.

[31] Muñoz-Espín D., Serrano M. (2014). Cellular senescence: from physiology to pathology. *Nature Reviews Molecular Cell Biology*.

[32] Turinetto V., Vitale E., Giachino C. (2016). Senescence in human mesenchymal stem cells: functional changes and implications in stem cell-based therapy. *International Journal of Molecular Sciences*.

[33] Brandl A., Meyer M., Bechmann V., Nerlich M., Angele P. (2011). Oxidative stress induces senescence in human mesenchymal stem cells. *Experimental Cell Research*.

[34] Macip S., Igarashi M., Berggren P., Yu J., Lee S. W., Aaronson S. A. (2003). Influence of induced reactive oxygen species in p53-mediated cell fate decisions. *Molecular and Cellular Biology*.

[35] Gire V., Dulic V. (2015). Senescence from G2 arrest, revisited. *Cell Cycle*.

[36] Krenning L., Feringa F. M., Shaltiel I. A., van den Berg J., Medema R. H. (2014). Transient activation of p53 in G2 phase is sufficient to induce senescence. *Molecular Cell*.

[37] Johmura Y., Nakanishi M. (2016). Multiple facets of p53 in senescence induction and maintenance. *Cancer Science*.

[38] de Vasconcellos J. F., Laranjeira A. B. A., Zanchin N. I. T. (2011). Increased CCL2 and IL-8 in the bone marrow microenvironment in acute lymphoblastic leukemia. *Pediatric Blood & Cancer*.

[39] Civini S., Jin P., Ren J. (2013). Leukemia cells induce changes in human bone marrow stromal cells. *Journal of Translational Medicine*.

[40] Ortiz-Montero P., Londoño-Vallejo A., Vernot J. P. (2017). Senescence-associated IL-6 and IL-8 cytokines induce a self- and cross-reinforced senescence/inflammatory milieu strengthening tumorigenic capabilities in the MCF-7 breast cancer cell line. *Cell Communication and Signaling*.

[41] Kletsas D., Pratsinis H., Mariatos G., Zacharatos P., Gorgoulis V. G. (2004). The proinflammatory phenotype of senescent cells: the p53-mediated ICAM-1 expression. *Annals of the New York Academy of Sciences*.

[42] Gorgoulis V. G., Pratsinis H., Zacharatos P. (2005). p53-dependent ICAM-1 overexpression in senescent human cells identified in atherosclerotic lesions. *Laboratory Investigation*.

[43] Jacamo R., Chen Y., Wang Z. (2014). Reciprocal leukemia-stroma VCAM-1/VLA-4-dependent activation of NF-*κ*B mediates chemoresistance. *Blood*.

[44] Konopleva M. Y., Jordan C. T. (2011). Leukemia stem cells and microenvironment: biology and therapeutic targeting. *Journal of Clinical Oncology*.

[45] Reikvam H., Brenner A. K., Hagen K. M. (2015). The cytokine-mediated crosstalk between primary human acute myeloid cells and mesenchymal stem cells alters the local cytokine network and the global gene expression profile of the mesenchymal cells. *Stem Cell Research*.

[46] Brenner A. K., Nepstad I., Bruserud O. (2017). Mesenchymal stem cells support survival and proliferation of primary human acute myeloid leukemia cells through heterogeneous molecular mechanisms. *Frontiers in Immunology*.

[47] Colmone A., Amorim M., Pontier A. L., Wang S., Jablonski E., Sipkins D. A. (2008). Leukemic cells create bone marrow niches that disrupt the behavior of normal hematopoietic progenitor cells. *Science*.

[48] Cheung L. C., Tickner J., Hughes A. M. (2018). New therapeutic opportunities from dissecting the pre-B leukemia bone marrow microenvironment. *Leukemia*.

