# Supplementary material for: Acute Leukemia Induces Senescence and Impaired Osteogenic Differentiation in Mesenchymal Stem Cells Endowing Leukemic Cells with Functional Advantages
Source: Stem Cells Int. 2019 Apr 1;2019:3864948. doi: 10.1155/2019/3864948 (PMC6466857; doi:10.1155/2019/3864948)
Supplement: Supplementary Materials — These experiments are related to the identification and characterization of MSC and to the effect on MSC of another leukemia cell line (SUP-B15) tested, to show that the results obtained are similar to the ones obtained with the leukemic REH cell line. Supplementary Figure 1: isolation and characterization of MSC. (a) BM-MSC immunophenotypic characterization by flow cytometry (dark grey histograms: isotype controls; antigen expression: light grey histograms). Cell surface markers and fluorochromes used are shown in each histogram. (b) Differentiation potential of BM-MSC; adipogenic differentiation showing lipid-rich vacuoles stained with Oil Red O (magnification 10x); osteogenic differentiation detected by ALP staining (magnification 10x); chondrogenic differentiation observed by Safranin O staining (magnification 10x). Left panels show control cells without induction. (c) Sphere-forming ability of BM MSC. A representative BM-MSC sample is shown. Supplementary Figure 2: cell growth and cell adhesion capacity of the ALL cell lines REH and SUP-B15. (a) The cell growth rate of REH and SUP-B15 cells was evaluated for 7 days at the different time points indicated after thawing fresh vials. (b) Percentage of adhesion of the REH and SUP-B15 cells to the MSC at 0, 1, 2, 4, and 6 h of coculture. Results are expressed as mean ± SEM (p values: nonparametric one-way ANOVA; ∗ p < 0.05 and ∗∗∗ p < 0.001). Supplementary Figure 3: SUP-B15 cells induce increased SA-β-Gal activity in MSC. (a) Representative micrographs (10x) of SA-β-Gal activity in MSC control (10% FBS) and MSC cocultured with SUP-B15 cells (SUP-B15 LN). Inset: magnification of the LN, showing absence of SA-β-Gal activity in SUP-B15 cells. (b) Quantification (%) of positive cells for SA-β-Gal activity in the different culture conditions (Student t-test, ∗∗ p < 0.01). (c) mRNA expression of p53 and p16 in MSC cultured in the SUP-B15 LN for three days. Results represent two independent experiments done in duplicates. R [file 3864948.f1.docx]

# Supplementary Materials: ACUTE LEUKEMIA INDUCES SENESCENCE AND IMPAIRED OSTEOGENIC DIFFERENTIATION IN MESENCHYMAL STEM CELLS ENDOWING LEUKEMIC CELLS WITH FUNCTIONAL ADVANTAGES.

Ximena Bonilla, Natalia-Del Pilar Vanegas, and Jean Paul Vernot.


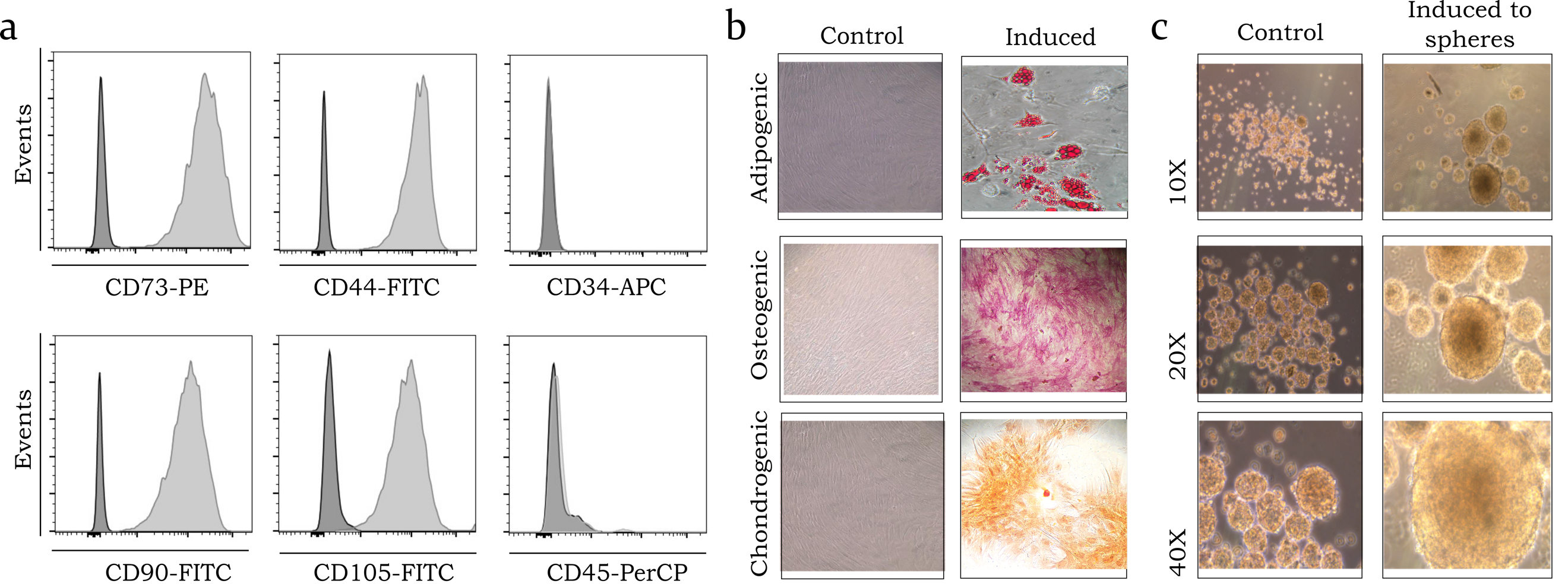


Supplementary Figure 1: Isolation and characterization of MSC. (a) BM-MSC immunophenotypic characterization by flow cytometry (dark grey histograms: isotype controls; antigen expression: light grey histograms). Cell surface markers and fluorochromes used are shown in each histogram. (b) Differentiation potential of BM-MSC; adipogenic differentiation showing lipid-rich vacuoles stained with Oil Red O (magnification 10X); osteogenic differentiation detected by ALP staining (magnification 10X); chondrogenic differentiation observed by Safranin O staining (magnification 10X). Left panels show control cells without induction. (c) Sphere forming ability of BM MSC. A representative BM-MSC sample is shown.


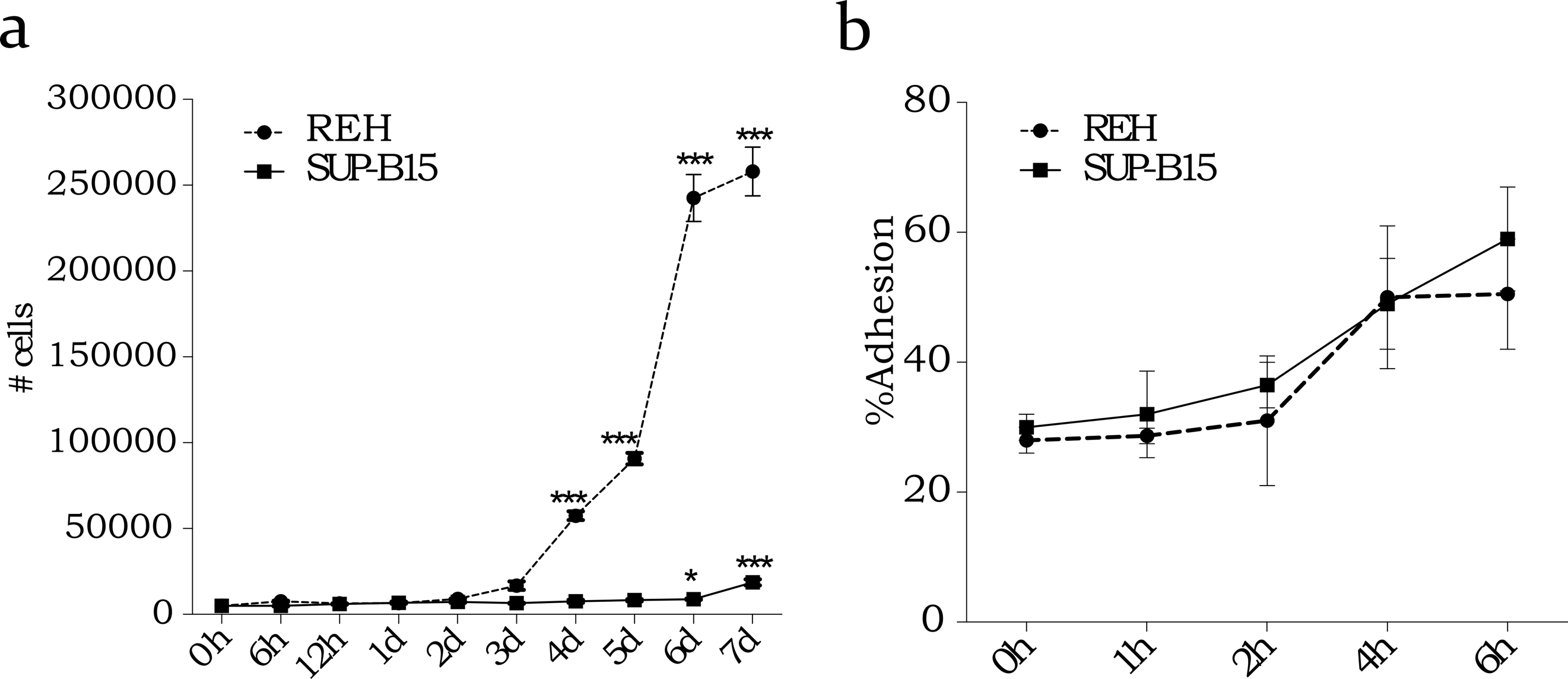


Supplementary Figure 2: Cell growth and cell adhesion capacity of the ALL cell lines REH and SUP-B15 (a) The cell growth rate of REH and SUP-B15 cells was evaluated for 7 days at the different time-points indicated after thawing fresh vials. (b) Percentage of adhesion of the REH and SUP-B15 cells to the MSC at 0, 1, 2, 4 and 6 h of co-culture. Results are expressed as the mean ± SEM (p values: non-parametric one-way ANOVA; *p < 0.05, ***p < 0.001).


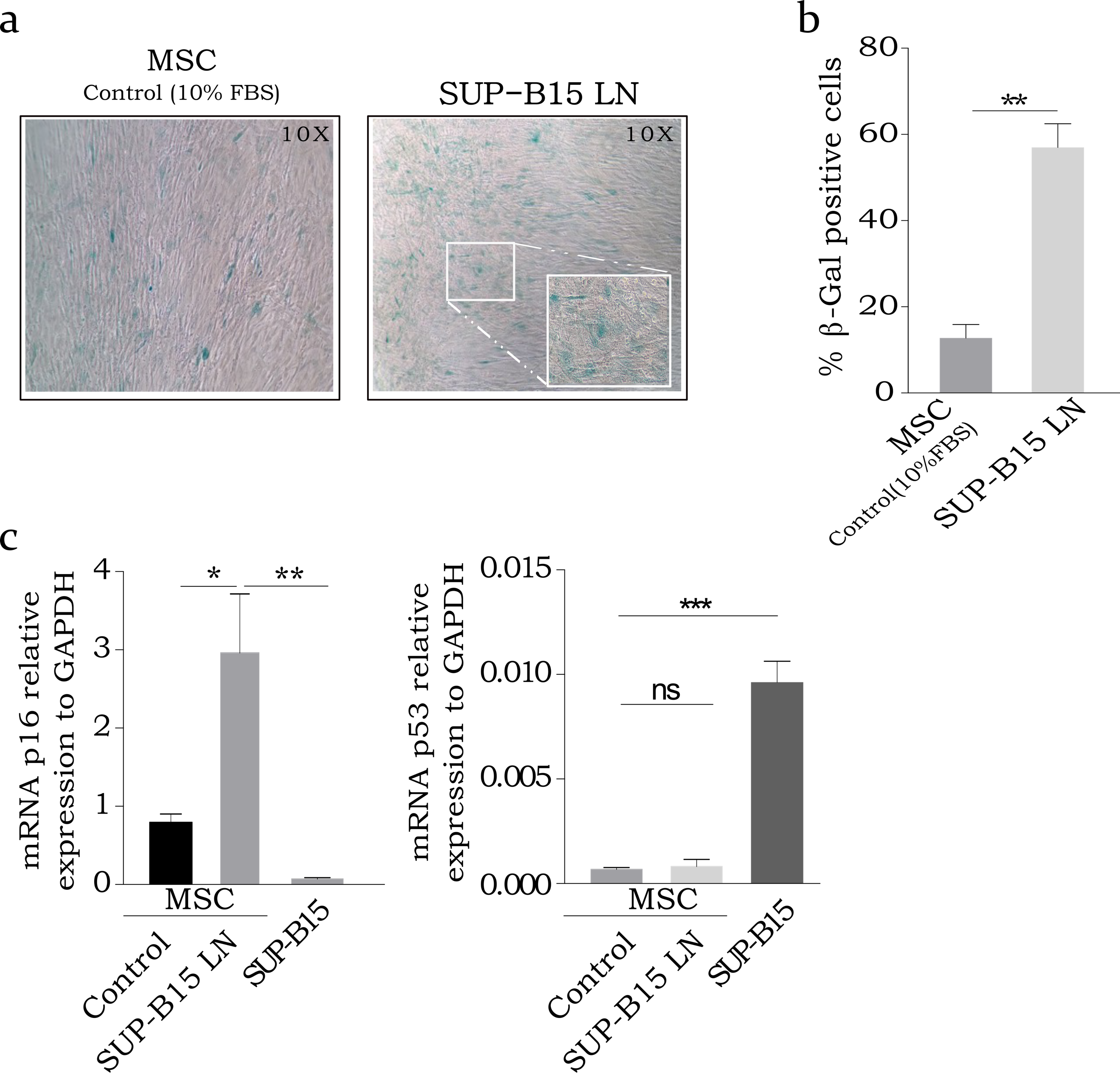


Supplementary Figure 3: SUP-B15 cells induce increased SA-β-Gal activity in MSC. (a) Representative micrographs (10X) of SA-β-Gal activity in MSC control (10% FBS) and MSC co-cultured with SUP-B15 cells (SUP-B15 LN). Insert: magnification of the LN, showing absence of SA-β-Gal activity in SUP-B15 cells. (b) Quantification (%) of positive cells for SA-β-Gal activity in the different culture conditions. (t-Student test, **p<0.01. (c) mRNA expression of p53 and p16 in MSC cultured in the SUP-B15 LN for three days. Results represent two independent experiments done in duplicates. Results are expressed as the mean ± SEM (p values: non-parametric one-way ANOVA; ns: non-significant, *p < 0.05, **p < 0.01, ***p < 0.001).


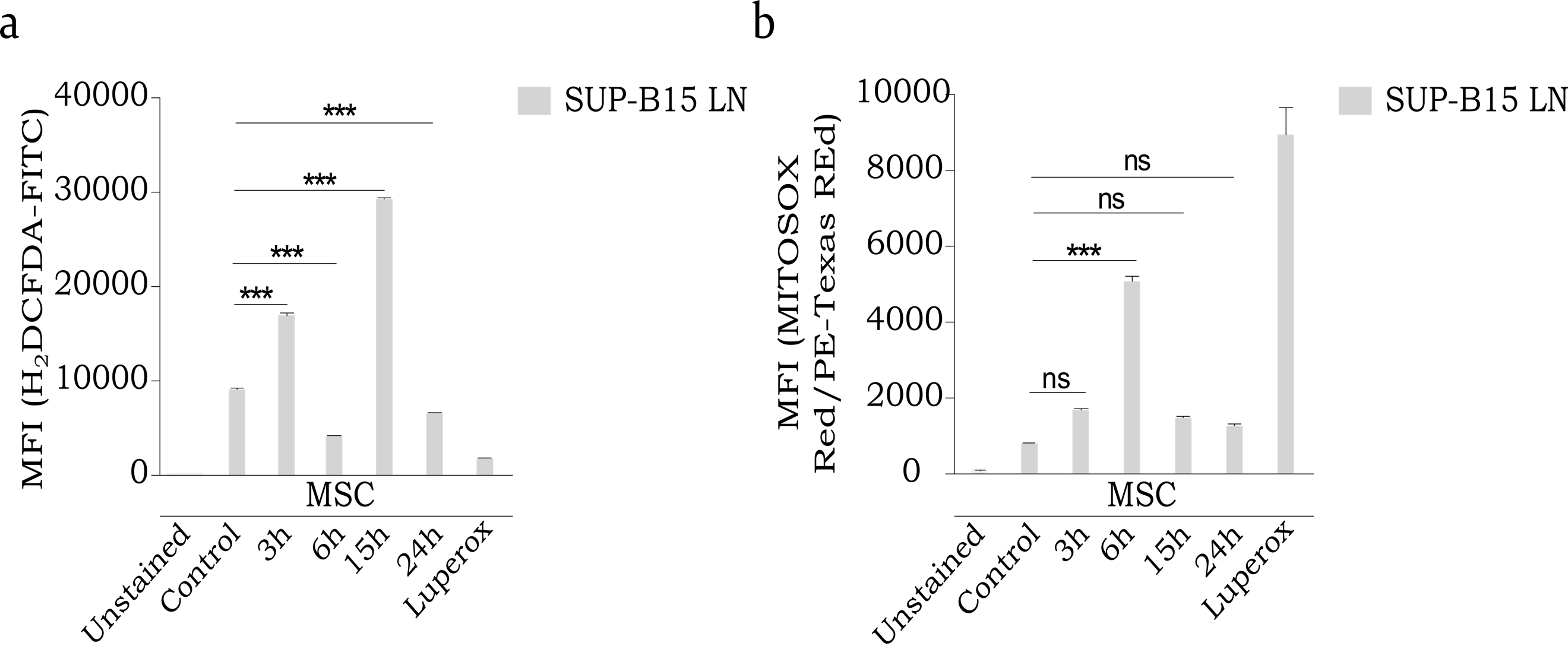


Supplementary Figure 4: Production of ROS in MSC co-cultured with SUP-B15 cells. (a) Mean fluorescence intensity of the cytosolic oxidative stress indicator H_2_-DCFDA in MSC of SUP-B15-LN and (b) Mean fluorescence intensity of MitoSOX RedTM (Mitochondrial ROS). Results are expressed as the mean ± SEM (p values: non-parametric one-way ANOVA; *p < 0.05, **p < 0.01, ***p < 0.001).
